# Selection and appointment of presidents of medical universities in Iran: Bridging reality and ideal through global and local evidence

**DOI:** 10.1371/journal.pone.0326563

**Published:** 2025-06-24

**Authors:** Leila Doshmangir, Mohammadhossein Somi, Sajad Lak, Neda Kabiri, Hossein Ebrahimipour, Omid Barati, Mohammadreza Amiresmaili, Mohammad Bazyar

**Affiliations:** 1 Department of Health Policy and management, Tabriz Health Services Management Research Center, School of Management and Medical Informatics, Tabriz University of Medical Sciences, Tabriz, Iran; 2 Social Determinants of Health Research Center, Tabriz University of Medical Sciences, Tabriz, Iran; 3 Department of Internal Medicine, Liver and Gastrointestinal Diseases Research Center, School of Medicine, Tabriz University of Medical Sciences, Tabriz, Iran; 4 School of Management and Medical Informatics, Tabriz University of Medical Sciences, Tabriz, Iran; 5 Research Center for Evidence-based Medicine, Iranian EBM Centre: A JBI Centre of Excellence, Faculty of Medicine, Tabriz University of Medical Sciences, Tabriz, Iran; 6 Medical Philosophy and History Research Center, Tabriz University of Medical Sciences, Tabriz, Iran; 7 Department of Health Economics and Management, School of Health, Mashhad University of Medical Sciences, Mashhad, Iran; 8 Clinical Research Development Unit, Shahid Haseminejad Hospital, Mashhad University of Medical Sciences, Mashhad, Iran; 9 Department of Healthcare Services Management, School of Health Management and Information Sciences, Shiraz University of Medical Sciences, Shiraz, Iran; 10 School of Management and Medical Informatics, Health in Disasters and Emergencies Research Center, Kerman University of Medical Sciences, Kerman, Iran,; 11 Health Management and Economics Department, Faculty of Health, Ilam University of Medical Sciences, Ilam, Iran; Thammasat University, THAILAND

## Abstract

**Background:**

The selection of university presidents who oversee health systems within their respective provinces has become increasingly critical in Iran, subjecting to an ongoing scrutiny and debate. This study aims to clarify the selection process and identify the essential competencies required for appointing medical university presidents both in Iran and globally, while also proposing relevant policy recommendations.

**Methods:**

This study employed a multi-method approach, incorporating a scoping review, document review, interviews, and brainstorming. The scoping review, conducted in September 2024, searching databases including PubMed, ISI Web of Knowledge, and Scopus. Interviews conducted from September 2024 to November 2024, were carried out with stakeholders from a diverse range, including university presidents, health ministers, healthcare managers, decision-makers, faculty members and key planners within the academic sector, whom were selected using purposive sampling method with maximum variation. A question guide was used, the main question of which included: essential characteristics and competencies of university president, prioritized criteria for decision making, feasibility of evidence-based decision-making, the process of selecting and appointing university presidents, and the strengths and weaknesses of this process. The data were analyzed using a framework analysis approach, informed by Modified Henry Mintzberg’s Management Model and a model of policy competency. Documents related to the selection and appointment of medical university presidents, including rules, regulations, and circulars, were collected through a census approach using both manual and electronic methods. Relevant documents were systematically retrieved from the official websites of the involved organizations. Brainstorming conducted in December 2024 and was used to develop policy recommendations.

**Results:**

Twenty six articles and 19 university protocols were included in the scoping review phase. Forty-two participants included in the qualitative phase of this study, most of who were president of a university. Also, 12 participants involved in the brainstorming phase. The responsibilities of a university president necessitating a blend of personal, interpersonal, professional and decisional competencies. In Iran, various factors including political preferences, lack of transparency and accountability, limited involvement from the university community, and insufficient emphasis on professional or technical competencies, influence the selection process of university presidents. The influence of some pressure groups including parliament representatives and the prevailing ideological climate within the Ministry of Health have varied across different governments, relegating essential professional competencies, such as the scientific credibility of the president, to low priority.

**Conclusions:**

During various selection and appointment periods, political changes and pressure groups’ interests have had a significant impact on the management changes in universities, and with governments’ changes, managerial capabilities are overshadowed by political tendencies. This study’s findings underscore the importance of transparency, evidence-based decision-making, and a systematic approach to the selection and appointment of presidents of medical universities. Implementing these insights can enhance the integrity of the selection process and improve governance in medical education in Iran.

## Introduction

Medical universities are complex organizations with the critical responsibilities of education, research, and health service delivery [1]. To achieve these objectives effectively, robust management and leadership are essential, particularly in light of the unique cultural, social, economic, and political contexts that shape each institution [2]. This is especially pertinent as we navigate rapid scientific and technological advancements. The university president plays a pivotal role in orchestrating these multifaceted management dimensions, rendering their leadership style profoundly influential across all aspects of university operations [3,4].

The selection and appointment of medical university presidents constitute a crucial process within both higher education and healthcare systems [5,6]. These decisions significantly shape the future of healthcare delivery and enhance the quality of medical education, with implications that extend Understanding the skills and qualities needed for university presidents and the right selection methods is highly significant nowadays, in the changing healthcare environment full of challenges [7]. Leaders who possess thinking crisis management skills and effective communication abilities are in demand. Successful university presidents display traits like problem solving skills, flexibility, foresight, hard work, political awareness willingness to take risks, proficiency, in managing change and strong networking capabilities [8]. Evidence emphasizes that behavioral coherence, the alignment between a leader’s words and actions, is vital for fostering mutual trust between university presidents and faculty members. He contends that faculty prefer presidents who embody the roles of defender, communicator, motivator, and facilitator, prioritizing leadership qualities over managerial attributes [7,9,10].

In recent decades, significant shifts have emerged in the financial landscape of higher education [11]. Increasing demands for cost transparency and financial efficiency, coupled with a diminished role of government funding for universities, have underscored the necessity for financial management competencies among university presidents [12]. These leaders must cultivate relationships with industry stakeholders and secure financial resources through external channels, including philanthropic donations [13]. As universities evolve towards greater responsiveness, autonomy, and freedom for faculty and staff, the imperative for effective leadership to unify individual efforts toward institutional goals has become increasingly pronounced [14].

Globally, universities face numerous challenges including rapid advancements in medical sciences and technology, the emergence of pandemics, and their subsequent impacts on healthcare systems [15]. Additionally, there is an urgent need for innovation in teaching and research methodologies amidst financial pressures [16]. Consequently, selecting competent presidents capable of navigating these challenges is essential [17].

In many developed countries, the selection of university presidents follows a multi-stage, transparent, and systematic process typically conducted by a committee comprising faculty members, staff, and student representatives based on clearly defined criteria for evaluating competencies [18]. In contrast, many developing countries including Iran struggle with these selection processes.

In 1985, the integration of the medical sciences sector into Iran’s higher education system, culminating in the establishment of the Ministry of Health and Medical Education (MoHME), significantly expanded the role of medical universities in the country [19]. These institutions, which specialize in medical and health-related disciplines, operate under the direct oversight of the MoHME. Their responsibilities extend beyond education to encompass critical functions such as governing public health, improving health standards within their provinces, conducting fundamental and applied research, and strategically managing the distribution of human resources and facilities. Furthermore, they are instrumental in facilitating access to medical services, securing financial resources, licensing medical practitioners, and ensuring compliance with national standards across educational, research, food and drug, health, and medical institutions. As of now, there are 69 medical sciences universities and schools in Iran.

The enactment of Article 49 of the Fourth Development Plan (2005–2009) [20] highlighted the imperative for universities to seek managerial and financial independence under the oversight of a board of trustees. This shift has heightened the demand for enhanced leadership and managerial skills within these institutions. It represents a move towards a quasi-local governance structure for each academic institution, establishing a management framework with distinct financial and employment regulations that symbolize greater organizational autonomy. In this evolving landscape, it is crucial to empower university presidents by augmenting their authority and responsibilities while ensuring they possess the requisite general and technical competencies.

Given the recent developments in education and research, alongside the diverse array of specialized roles confronting medical universities within healthcare systems, challenges such as financial constraints, rising healthcare costs, and the optimization of resource utilization and human resource management have become increasingly pressing [21]. Addressing these challenges through the strategic selection of university presidents is critical to enhancing institutional performance and, by extension, improving healthcare service delivery. Accordingly, this study aimed to examine the selection processes and identify the core competencies required for appointing medical university presidents in both Iran and international contexts, while also offering policy recommendations to support evidence-informed decision-making.

## Methods

This multi-method study was conducted in three phases: a scoping review, document review and qualitative interviews, and brainstorming through an expert panel.

### Phase 1: Scoping review

Since we desired to assess comprehensively about characteristics of university presidents and their selection criteria, a scoping review was preferred. This phase was conducted in September 2024. For the scoping review phase, we adhered to the Joanna Briggs Institute (JBI) methodology [22,23], and followed the Preferred Reporting Items for Systematic Reviews and Meta-Analyses extension (PRISMA-ScR) [24]. In this scoping review the inclusion criteria were defined as follows: P (Population): university presidents/chancellors; C (Concept): selection process of university presidents and the competencies required for them; and C (Context): publications related to universities worldwide. Studies were excluded if there were not any relevant data to be extracted. Studies focusing on other academic leadership roles such as deans and department heads and studies that do not address the selection process or competencies, such as papers focusing only on university rankings, funding, or student outcomes were excluded. Studies without extractable or relevant data and studies focusing on leadership in non-academic sectors were also excluded.

We conducted comprehensive searches using both free-text keywords and controlled vocabularies across several databases without publication date restrictions, including PubMed, ISI Web of Knowledge, Scopus, and PsycINFO (via Ebsco). The main keywords included university, school, college, selection, recruitment, appointment, president, leader, chancellor, competency, and skill. A full search strategy of all the above mentioned databases are provided in S1 Table. Also, we searched Google Scholar for further relevant literature. The first 100 results were screened. These 100 were chosen because of data saturation, researchers found that studies after 100 results, were not relevant to the topic. To enrich our findings, we also reviewed the web pages of top universities in nine countries including USA, Canada, England, Sweden, Japan, Turkey, Germany, Austria, and the Netherlands, which were selected purposefully to represent a variety of selection processes. These countries were chosen based on the accessibility and availability of information regarding their presidents’/ chancellors’ selection processes on their official websites. A list of the universities examined is provided in S2 Table.

**Fig 1:**
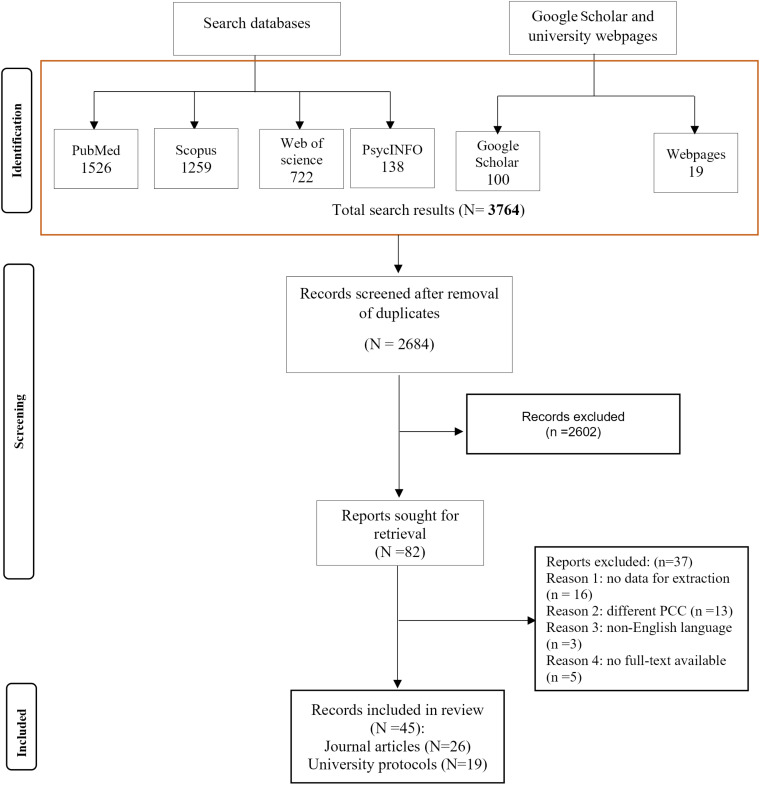
PRISMA flow diagram of study selection in the scoping review phase.

The searches were conducted in September 2024, focusing exclusively on English-language publications. The search results were imported into EndNote X.20 (Clarivate Analytics, PA, USA) for study selection, which was carried out by two independent reviewers based on the established inclusion and exclusion criteria. Data extraction was also performed by two independent reviewers using a standardized data extraction sheet aligned with the recommendations from the JBI methodology group for scoping reviews. To summarize the available evidence concerning the research question, we employed qualitative deductive content analysis [25,26]. We utilized Henry Mintzberg’s managerial roles model [27] and a model of policy competency [28] to categorize the extracted codes. Mintzberg’s management model highlights the dynamic nature of management by categorizing managerial roles into three primary areas: interpersonal, informational, and decisional. The model of policy competency outlines a set of competencies-abilities or skills-emphasizing three core competencies: analytical, operational and political competencies. The flow diagram of selecting studies across various databases and websites is illustrated in Fig 1.

### Phase 2: Qualitative interviews and document review

In this phase, qualitative individual interviews and a document review were conducted to gather data regarding the selection processes and competencies required for appointing presidents of medical universities in Iran. This phase was conducted concurrently with the previous phase, from September 2, 2024 to November 10, 2024.

#### Data collection.

Interviews were conducted with 42 key stakeholders, including health ministers, university presidents, managers across different levels of the health system, faculty members, and other relevant participants. Individuals were selected for interviews if they had experience serving as the president of a medical university or faculty, or had been involved in the process of selecting and appointing university presidents. Semi-structured interviews provided an open environment for respondents to share their experiences and perspectives (see S3 File for interview guide). Purposive sampling with maximum variation was employed to ensure the inclusion of individuals with diverse yet relevant expertise and knowledge. Each interview lasted between 60 and 110 minutes, was recorded with the interviewees’ informed consent, and subsequently transcribed. In addition, documents such as rules, regulations, and circulars related to the selection and appointment of medical university presidents were collected through a census approach, both manually and electronically, from all relevant organizations. The official websites of these organizations were also systematically searched to identify and retrieve pertinent documents.

#### Data analysis.

The data were analyzed using a framework analysis approach, informed by Modified Henry Mintzberg’s Management Model [27] and a model of policy competency [28]. This structured approach enabled the systematic identification of key themes and sub-themes. The analysis followed several interconnected stages. First, a process of familiarization was undertaken in which interview transcripts were read carefully and repeatedly to gain an in-depth understanding of the data. Next, key concepts and themes were identified and coded. These initial codes were then organized into broader categories, forming an analytical framework comprising overarching themes and related sub-themes. Finally, the themes were analyzed and interpreted in relation to the study’s research questions, allowing for a coherent and meaningful understanding of the findings. To ensure the rigor and credibility of the findings, several strategies were employed. The diversity of the sample was enhanced by including participants from different levels of the health system. The findings were presented to colleagues for feedback, and data were cross-validated through triangulation with additional participants and secondary sources. All stages of the research were meticulously documented to enhance transparency and reproducibility. These measures were taken to ensure the validity and reliability of the study findings.

### Phase 3: Brainstorming

In the final phase, conducted in December 2024, a brainstorming session was held to develop policy recommendations. During this expert panel, the results from previous stages,both local and global evidence, were presented to the participants. They were asked to discuss the issues and provide policy recommendations regarding the required competencies for university presidents and the appropriate processes for their selection.

The participants (n = 12) were selected using purposeful sampling. Through a process of discussion and collaboration, an initial set of 13 policy recommendations was developed concerning the appropriate processes for selecting university presidents in universities. These recommendations were subsequently revised and refined, resulting in four key policy recommendations. Based on criteria such as comprehensiveness, implementation capability, and potential impact, seven main policy recommendations were ultimately identified. These recommendations aim to enhance the selection process and competency framework for university presidents in medical universities in Iran.

**Fig 2:**
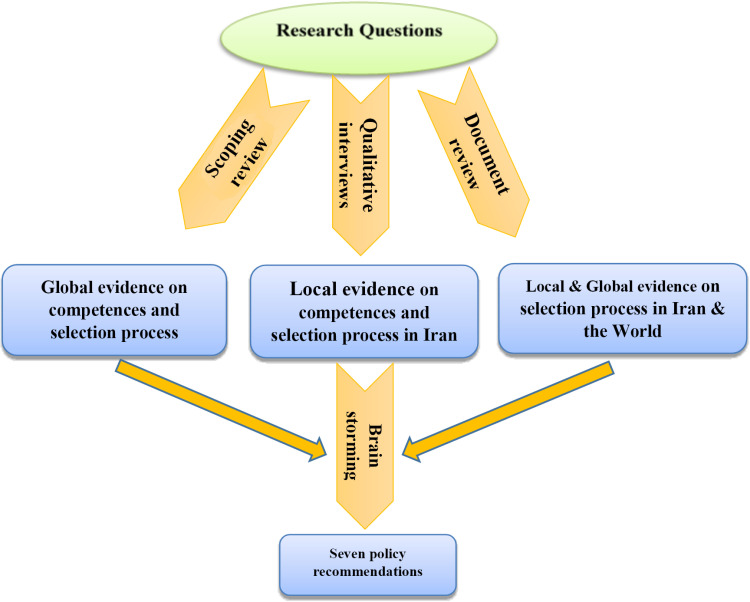
Schematic diagram of the research phases.

A schematic diagram of the research phases is indicated in Fig 2.

### Ethical considerations

This study was approved by the ethics committee of Academy of Medical Sciences of Islamic Republic of Iran (Approval No: IR.AMS.REC.1403.009) and Tabriz University of Medical Sciences, Tabriz, Iran (Approval No: IR.TBZMED.REC.1402.211). The written informed consent was obtained from all participants in the second and third phase of the study. Also, participants were assured to be anonymized.

## Results

The results are organized according to the main phases of the study: scoping review, qualitative analysis (interviews and document review), and brainstorming session.

### Phase 1: Findings of the scoping review

#### Study selection and characteristics of included studies:

A total of 26 journal articles and 19 university protocols concerning the selection processes and competencies for university presidents were included in this scoping review.

Of the included studies, 25 were conducted in the following countries: the USA [29–42], Iran [43–45], Canada [46], Belgium [47], Uganda [48], Jordan [49], Afghanistan [50], Korea [51], China [52], and the UK [53]. One study provided a global review [54]. The publication dates of these studies ranged from 1994 to 2021, with six specifically focusing on medical schools [30,36,37,39,43,48]. See S4 Table for the main characteristics of the included studies.

#### Main findings:

**Required competencies for university presidents:**

The competencies required for university presidents were categorized according to two frameworks [27,28]: personal, interpersonal, professional, and decisional competencies.

1. Personal competencies

Key personal attributes that enhance a university president’s effectiveness as a leader include adaptability, self-awareness, and resilience.

2. Interpersonal competencies

Effective interpersonal competencies and communication skills are crucial for university presidents. These skills encompass the ability to convey messages clearly, accurately interpret incoming information, and foster productive personal interactions in diverse situations. As detailed in Table 1, the primary interpersonal competencies identified for university presidents include leadership, negotiation, and communication skills.

**Table 1:** Main competencies required by the university presidents (results of the scoping review phase).

Main categories	Sub-categories	Competencies
Personal competencies	Adaptability	- Revolutionary - Time management skills - Responsible and conscientious - Recruitment and retention of talent - Managing stress - Using power wisely - Punctuality - Role-model - Full recognition of duties - Mentoring - Accountability
	Self-awareness	- Knowing self - Personnel Management - Does not take self too seriously - Adherence to moral principles - Open to Learning and self- improvement - Continuous individual development - Learns from self-reflection - Learns from others
	Resilience	- Inclusivity - Courage - Strength and wisdom - Cultural competence - Demonstrates resourcefulness - Powerful - Well mannered - Being energetic - Ability to concentration - Ability to control personal conduct - Decisiveness - Admitting errors - Openness - Possess perseverance
	Emotional intelligence	- Candour and honesty - Trustworthiness - Interactive - Attractiveness - Critic - Authenticity - Respect - Integrity - Behavioral coherence - Commitment - Maximizing values
Interpersonal competencies	Leadership	- Social intelligence - Seeks to understand human behavior in multiple contexts - The need to consult others - Incorporate a culture of teambuilding - Create mutual trust - Ability to work with others - Communication with diverse audiences - Presents self professionally as a leader - Demonstrates unselfish leadership - Support leadership of others - Lead by example - Lead the team of executive officers - Serve as the “face” of the institution - Recruit key leadership - Being visible - An inclusive style of leadership - Ability to build trust - Ability to influence and motivate people - Ability to build consensus - Ability to demonstrate consistent judgment - Ability to maintain core values
	Communication	- International Culture, Vision, and Mind set - Have overseas experience - Expresses views articulately in multiple forms of communication - Possess strong written communication skills - Verbal communication skills
	Negotiation	- Serve as a political lobbyist - Connection with government or industry - Communication with sponsors - Improving relations with the teaching hospitals - Demonstrates negotiation skills - Ability to bargain and convince the other party - The ability to listen, negotiate and persuade - Liaising - Open discussion of complex or awkward topics
Professional competencies	Specialized knowledge	- Being as a tenured full professor - Academic competence - Academic credibility - Medical expert - Medical educator - Being outstanding in terms of science - Academic management experience - A history of success in previous jobs - Having doctoral degree - Professional maturation - Knowledge of academic life - Knowledge of the “academic coal face” - Knowledge of academic processes - Understanding the university environment, structure, and function - Research skills - Futuristic - Practicability - Familiarity with management and leadership theories in higher education - Knowledge of university standards
	Specific technical skills	- Technical expertise - Effective use of information technology - Work effectively with the media - Familiarity with English language in management
	Knowledge of rules and regulations	- Having legal skills - Knowing laws and adhering to the implementation of laws - Familiarity with rules and regulations - Adherence to ethical principles
Decisional competencies	Logical reasoning	- Strategic planning - Responds appropriately to change - Communicates vision effectively - Act strategically - Improving the innovation - Foster the development and creativity of learning organizations - Give and receive constructive feedback - Create coalition and change support - Engage multiple perspectives in decision making - Political sensitivity - Institutional acumen - Strength to stand up to political pressure - System thinking - Critical thinking - Analytical thinking - Focus on implementing reforms in practice - Understanding effective and up-to-date management practices - Commitment to delegate authority - Environmental analysis skills (SWOT analysis)
	Disturbance Handling	- Accurately assesses the costs and benefits of risk-taking - Crisis management - Ability to diplomatically engage in controversial issues - Responsiveness to organizational goals - Management stability - Institutional assessment - Organizational capability - Ability to make and stand by tough decisions - Effectiveness as a departmental and institutional administrator - Navigating Challenges
	Resource Allocation	- Fundraising - Financial stewardship - Financial understanding - Financial acumen - Administrative acumen - Access to outside resources and an ability to use them

3. Professional competencies

Professional competencies are essential for equipping university presidents for success within their roles and for ongoing career management. These competencies pertain specifically to the university context and the health system. Based on our analysis, the core professional competencies required include specialized knowledge, specific technical skills, and an understanding of relevant rules and regulations.

4. Decisional competencies

Decisional competencies are vital for university presidents to effectively manage tasks, resources, and information, thereby achieving objectives and maintaining productivity within the complex environment of a university. As shown in Table 1, subcategories of decisional competencies encompass logical reasoning, resource allocation, and disturbance management.

**Selection process of university presidents:**

The selection process for university presidents has been elucidated in eight studies and nine university protocols. Findings from these studies indicate that university presidents are appointed through various methods, including: 1) Analytical Hierarchy Process (AHP); 2) job advertisements; 3) the Helios Web-based open-audit voting system; 4) elections by faculty members; and 5) search and selection committees.

1. Analytical hierarchy process (AHP)

The Analytical Hierarchy Process (AHP), as described by Gibney et al. [29], involves selecting a university president based on thirteen leadership criteria. These criteria encompass interpersonal and environmental skills, vision, resource accessibility and utilization, as well as the requirement for candidates to hold the position of tenured full professor at the university. The selection committee typically comprises alumni representatives, members from the Office of the Provost, and representatives from affiliated research centers.

AHP is a robust decision-making technique that facilitates evaluations based on multiple criteria. It effectively captures subjective assessments, including emotions, ideas, and sentiments. The outcome of the AHP process is a ranked list of preferences for various options, with cardinal values reflecting not only the order of preference but also the degree to which one option is favored over another.

The AHP methodology comprises three essential steps: decomposition, comparative evaluation, and priority synthesis.

- Decomposition: This initial step involves creating a hierarchical structure that delineates the decision problem. The main objective is positioned at the top, followed by criteria, sub-criteria, and alternatives arranged in descending order.
- Comparative evaluation: In this phase, the decision-maker constructs matrices at each hierarchical level by comparing pairs of elements. At higher levels, criteria and sub-criteria are assessed based on their significance, impact, or contribution to the overall goal. At the lowest level, alternatives are compared concerning each criterion or sub-criterion directly above them, referred to as their covering criteria.
- Priority synthesis: This final step synthesizes the results of the comparisons to derive a comprehensive ranking of options.

By employing AHP, universities can ensure a systematic and transparent approach to selecting their leaders, ultimately enhancing the efficacy and credibility of the selection process [29].

2. Job advertisements

According to Lavigne et al. [46], some universities in Canada use job advertisements to recruit candidates of university chancellor based on predetermined qualifications. The advertisements are published in the ‘University Affairs magazine’ and the ‘Canadian Association of University Teachers bulletin’.

3. Helios Web-based open-audit voting system

In this method, which was used in Adida’s [47] study, university president was selected by all members of the university, including faculty, students, and staff using Helios, a web-based open-audit voting system.

4. Election by the faculty members

In the qualitative study by Kyamanyawa et al. [48], is reported that in the public medical schools, faculty members and senior academic staff, elected university president and the University Council appointed their result. The university president tenure is for four years and can only serve for a maximum of eight years. On the other hand, in the private schools, presidents were sourced and appointed by the proprietors or the university leadership.

5. Search and selection committee

This method was used in the study by Enomomto et al. [31], where the search committee for a new dean was chaired by an existing dean and included several faculty members. Similarly, Harvey et al. [54] noted in their review that the central administration first convened a dean selection committee, which subsequently conducted a SWOT analysis to identify the school’s needs. Candidates were initially screened via phone interviews, followed by in-person interviews where they presented to the faculty, engaged with external advisory groups, and met with representatives from central administration. The final assessment of candidates culminated in a somewhat confidential voting process.

In the review by Yi et al. [51], it was reported that university presidents in South Korea were elected directly from 1988 to 2005, transitioning to an indirect election system-utilizing simple random sampling- since 2005. This latter approach incorporated methods such as random drawing and systematic sampling to ensure a representative selection of all members.

Conversely, Liu et al. [52] highlighted a lack of democratic engagement in the university selection process in China, where many faculty members felt excluded and information was often insufficiently transparent. Furthermore, the educational philosophies of presidential candidates were not rigorously evaluated during the selection process. Notably, the tenure of university presidents in China is relatively short, with studies indicating an average duration ranging from 3·5 to 9·5 years.

A comparative overview of the selection processes of 19 university protocols across nine countries is presented in Table 2.

**Table 2:** Selection process of universities presidents in 19 universities across 9 countries.

Country	University president selection process
USA	The president is the leader of the university and its board. The university’s board of trustees appoints the university president through its 20-member Presidential Search Committee, which includes representation from faculty, students, staff, and the Corporation.
Canada	About 18 months before the end of the President’s term of office, or at any time a vacancy is to be filled, a committee shall be formed to advise Governing Council on the appointment of a President.
England	Congregation, the sovereign body of the University, which acts as its ‘parliament’, is responsible for electing members to Council and other University bodies, and approving the appointment of the Vice-Chancellor. The Chancellor is usually an eminent public figure elected for life, serves as the titular head of the University, presiding over all major ceremonies.
Sweden	The election committee decides about the president. Steps of recruitment of a new president: 1: Final candidates are presented to the Consultative College and the Consultative College meets. 2: The Election Committee meets and decides who to put forward as a candidate for KI president to the University Board. 3: The University Board decides which candidate should be proposed to the government as the new KI president. 4: The University Board decides on the process for the appointment of KI’s vice president. 5: KI’s new president takes office
Japan	A joint meeting is established for the Selection of the Head of the University. The Selection Meeting decides on the selection criteria, term of office, and selection methods for the head candidate of the University in Tokyo, and conducted the selection process. The head’s term of office is four years.
Turkey	The Rector is the academically and administratively senior executive of Turkey’s University and represents the legal entity of the University externally. The Rector is primarily authorized and responsible for the rational use and development of the teaching capacity of the University and its affiliated units, the provision of necessary social services to students, the taking of security measures when necessary, the planning and execution of education, scientific research, and publication activities, and the conduct of scientific and administrative supervision. The Rector of a state university is appointed by the President of the Republic of Turkey. In addition to the Rector, the rectorate includes vice rectors. The Rector chooses a maximum of three vice-rectors among the professors of the University to assist him in his work. The vice-rectors’ term of office is limited to the Rector’s term of office.
Germany	The Senate is the highest decision-making body of the university. As part of the University Act, it decides on many of the principles of the Universities in German, such as the election of the president and the long-term development of the university. The Senate is made up of teachers, academic staff, students and technical and administrative staff in a ratio of 6:2:2:1. The Senate is a decision-making body. To prepare decisions, it therefore sets up various thematically defined committees in which members of the university deal with issues in terms of content.
Austria	The university management in Austria consists of Rectorate, Senate and University Council. The Rectorate governs the University. It consists of a Rector and four Vice-Rectors for the Research and Innovation, Finance, Clinical Affairs and Education Divisions. The Senate is made up of 13 representatives from among the University’s professors, six representatives of university teaching and research staff, one representative of the general university staff and six student representatives, either elected or appointed. The term of office of the Senate is three years. All persons who are in active employment or service at the University on the day of announcement of the elections (“effective date”) are eligible to vote and to stand for election. Proposals for election may be submitted in writing to the election commission no later than two weeks before the election day. Like the Senate, the University Council is a central governing body. Two of the Council’s members are appointed by the Senate of the University, and two by the federal government. A fifth member is elected by these four members. University Council members hold office for five years.
Netherlands	The Executive Board is responsible for the efficient and effective management of the Universities in Netherlands. The Executive Board is the highest administrative body of the University. The Board of Governors appoints, suspends and dismisses the members of the Executive Board. The Executive Board regularly consults the University Council, which advises the Executive Board and has a say in important matters and decisions. The Executive Board and the faculty deans consult in the Administrative Meeting, and the deans also have their own consultation structure: the Council of Deans, which is chaired by the Rector Magnificus.

### Phase 2: Findings of the qualitative study

**Fig 3:**
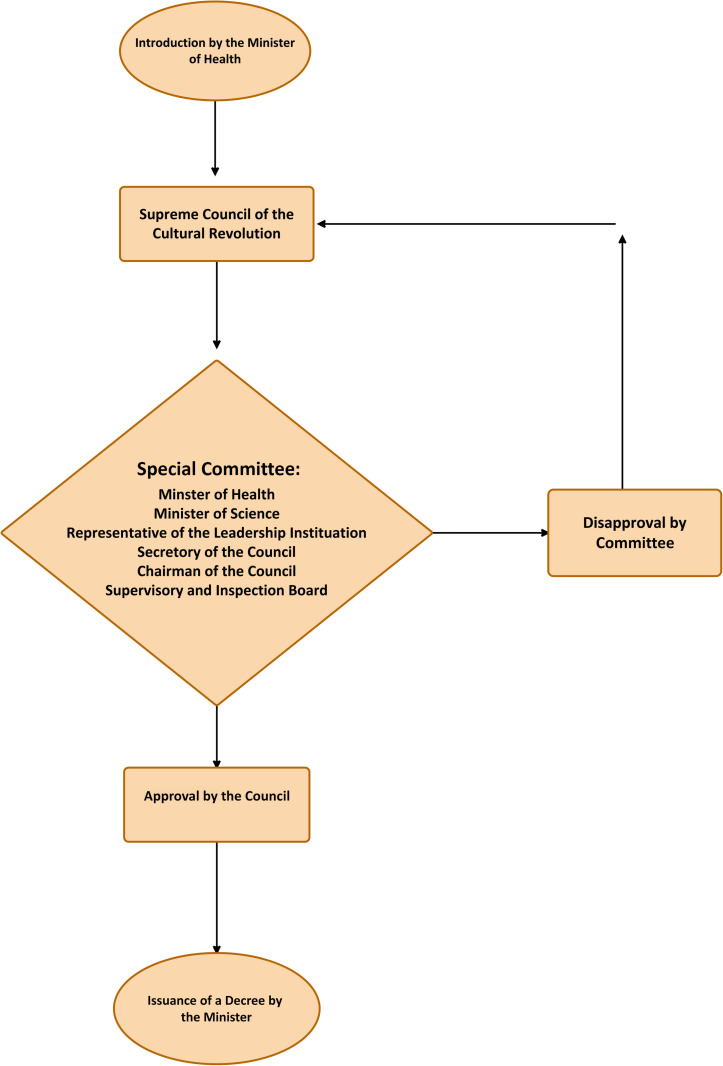
Flowchart of the selection process for medical university presidents in Iran.

Fig 3 illustrates the selection process for medical university presidents in Iran.

Forty-two participants included in the qualitative phase of this study, the main characteristics of whom are indicated in Table 3. This qualitative study, grounded in document review and stakeholder interviews within the Iranian health system, presents key findings in two primary areas: the essential competencies for medical university presidents and the processes involved in their selection and appointment.

**Table 3:** Main characteristics of participants in the qualitative interview phase of this study.

Organizational position	Gender		Number
	Female	Male	
Minister of Health	2		2
President of the university	1	20	21
Faculty member	2	8	10
Hospital manager	1	2	3
High-level employees of the Ministry	1	2	3
Post graduate students	2	1	3
Total number of participants			42

**Required competencies for university presidents:**

The competencies necessary for effective leadership in medical universities are categorized into four main domains: personal, interpersonal, professional or technical, and decisional (Table 4). This framework encompasses a total of 28 sub-themes that further delineate the specific skills and attributes required.

**Table 4:** Main competencies required by the university presidents in Iran (results of the qualitative phase).

Themes	Sub-themes	Abilities and skills/ competencies
Personal competencies	- General qualifications - Ethical and behavioral skills - Self-managing - Unraveling complex challenges - Building bridges for change - Crisis management and resilience Building - Willingness to learn	- Self-awareness - Self-control and regulation - Flexibility - Open-mindedness - Emotional intelligence - Resilience - Adapting to change - Ability to recognize personal strengths and weaknesses - Acceptance of constructive criticism - Positive thinking and energy - Generation - Cognitive abilities - Innovative thinking - Critical and analytical thinking - Futuristic visioning - Stress-reduction skills - Ability to work under pressure - Strategic thinking and Planning
Interpersonal competences	- Supportive work environment - Client-centered approaches - Building leadership - Partnerships and alliances - Empowering people - Negotiation - Cultural awareness - The power of teamwork - Empathetic engagement - Knowledge sharing	- Establishing effective communication - Inter-sectoral leadership - Understanding of the characteristics of the region - Influence and impact capacity - Team building and collaborative skills - Management of conflicts of interest - Negotiation and persuasion Skills - Stakeholder Engagement and participation - Stakeholders’ analysis
Professional competencies	- Previous work experiences - knowledge of rules and regulations - Knowledge of university domains ‘duties - Knowledge of specialized issues in medical university - Continuous professional development	- Knowledge of specific technical skills - Understanding of the health system - Knowledge of university standards - Knowledge of research topics and discussions - Familiarity with educational Topics and discussions - Familiarity with administrative and financial matters - Familiarity with higher education development priorities - Familiarity with developments’ plans - Proficiency in international communications and interactions - Experience in health system - Management, policy-making, and health economics - Project management
Decisional competencies	- Data-driven decision-making - Alternatives development - Policy evaluation - Political awareness - Good governance - Management knowledge and skills	- Environmental change and interpretation - Problem-solving abilities - Foresight and predictive analysis - Systems thinking - Systems thinking and holistic analysis - Development and empowerment initiatives - Prioritization and resource allocation

1. Personal competences

Interviewees consistently emphasized the paramount importance of personal competencies in the selection of university presidents. Critical traits identified include flexibility, ethical conduct, anger management, problem-solving skills, a positive attitude, and self-awareness. Participants also highlighted loyalty, self-management, and professional knowledge as vital indicators of a candidate’s suitability for the role of university president.

According to national regulations, particularly the Comprehensive Regulations for the Management of Universities, the selection process for university presidents is governed by both general and specific criteria. General conditions mandate adherence to religious obligations, commitment to the Constitution, and a strong reputation across ethical, academic, and cultural dimensions. Specific conditions stipulate that candidates must hold at least an associate professor rank and possess a minimum of five years of management experience in academic or scientific roles. However, these regulations have faced criticism for inadequately capturing the essential leadership qualities required for university presidents.

Participants advocated for a thorough assessment of personal competencies prior to candidate selection. One interviewee remarked, “*Personal competencies should be evaluated through various methods. Key competencies such as disaster management, stress management, and change management must be assessed before selection*.” (P11)

Moreover, it was emphasized that both the university president and their accompanying team should be individuals capable of introducing innovative ideas and initiatives into the institution, moving away from outdated practices that hinder progress.

1. Interpersonal competencies

Participants emphasized that an effective university president must possess strong communication skills and engage in scientific, humane, and precise interactions with staff. Additionally, they should be adept in motivational and incentive systems, applying these effectively to advance the university’s core mission.

A recurring theme among interviewees was the critical importance of effective communication and interpersonal skills. This includes not only engagement with the media but also continuous dialogue with subordinates and collaboration with board members, all of which are essential for enhancing accountability within the institution.

Moreover, presidents must demonstrate political acumen, courage, and robust advocacy skills, enabling them to effectively defend the university’s mission during negotiations with external agencies and relevant government officials. The capacity to cultivate constructive relationships with government entities, legislators, the public, professional associations, and the medical community is crucial. Such connections can facilitate access to additional resources and support.

Many interviewees underscored the significance of a university president’s familiarity with the regional context in which the university operates. As one participant noted, “*The university president must have a comprehensive understanding of the culture, language, and customs of the region served by the university. It can be said that being indigenous is considered an advantage*” (P40).

Furthermore, the ability to communicate effectively with community leaders, local governors, and organizations such as the Ministry of Health and Medical Education (MoHME) exemplifies essential cross-sector leadership and inter-organizational communication skills. One participant articulated this necessity: “*To fulfill one of their primary duties-communication-the university president must develop a clear strategy for managing diverse stakeholders*” (P21).

These insights collectively highlight the vital interpersonal competencies required for successful leadership in medical universities, emphasizing the need for a president to navigate complex social and political landscapes while fostering collaborative relationships.

1. Professional competencies

An analysis of the competencies required for university presidents reveals that the existing criteria established by governing bodies are overly broad and insufficiently focused on the specific professional skills necessary for effective leadership. Currently, these criteria primarily emphasize general qualifications, such as a minimum of five years of experience at the assistant professor level or higher, with some exceptions allowing candidates with as little as three years of experience. However, these general stipulations fail to encompass the comprehensive range of professional competencies essential for effective university management.

As one interviewee poignantly noted, “*I regret to say that during this period, we have witnessed the appointment of individuals as university presidents who lack even basic managerial experience. This oversight will undoubtedly inflict serious harm on the university’s structure*” (P31).

The intricate dynamics of a system that integrates both healthcare and medical education necessitate that university presidents possess specialized professional and technical competencies. Interviewees underscored the importance of university leaders taking primary responsibility for health-related missions, which are deeply intertwined with social, economic, and governance issues. Furthermore, presidents must be well-versed in national and international regulations that underscore a commitment to ethical and academic standards. Aligning their responsibilities with these broader concerns is paramount. One participant articulated this need: “*As universities encompass various fields such as healthcare, education, and research, a president must have a comprehensive understanding of all these domains*” (P34).

Many interviewees expressed a preference for university presidents to have academic backgrounds or to complete educational programs in health system management, policy-making, or health economics. They contended that familiarity with management principles, health economics, epidemiology, and governance is crucial for effective university administration. Adopting a holistic, community-oriented approach enables university presidents to prioritize public health initiatives and engage stakeholders in advancing the health system.

Additionally, the importance of ongoing training post-appointment was emphasized. It is essential for presidents to identify diverse stakeholders, including benefactors, and to understand the financial mechanisms underpinning university operations. Familiarity with the roles of governing bodies such as the council, board of trustees, and board of directors is equally critical for effective leadership.

1. Decisional competencies

Experts widely agree that a university president must strategically leverage policy to prioritize health issues, ensuring that health does not become a mere instrument for political maneuvering by legislators, special interest groups, or rent-seeking entities. As one expert noted, “*A university president should possess political acumen. This does not necessitate a Ph.D. in political science or public policy; rather, it entails exercising political power within a public sector environment characterized by conflicting interests*” (P36).

Higher-level governance documents delineate the extensive responsibilities of university presidents, which encompass management, planning, organization, and the formulation of executive policies. These duties span a broad spectrum of activities—scientific, educational, cultural, social, and administrative. University presidents must adeptly coordinate and oversee the implementation of these policies, appoint faculty and student council members, and advocate for the university’s interests in legal matters. The capacity to navigate these multifaceted roles is essential for fostering the university’s active engagement within society.

As one interviewee remarked, “*Some changes arise from the resignation of an individual or their inability to achieve managerial satisfaction. Nevertheless, there is a significant degree of stability in management, as leaders recognize that neglecting established management frameworks will lead to inevitable change*” (P14).

A recurrent theme among interviewees was the necessity of evidence-based decision-making. One participant highlighted a critical weakness observed in many university presidents: “*A significant number fail to make decisions grounded in scientific and credible evidence. Instead, their choices are often influenced by a narrow perspective limited to their own experiences and those of their immediate circle. Decisions should be informed by comprehensive feedback from the broader environment and supported by reliable data*.” (P26)

In summary, the ability to make informed, evidence-based decisions is not only crucial for effective leadership but also imperative for advancing the university’s mission in an increasingly complex and interconnected world.

**Selection and appointment process of university presidents:**

The process of selecting and appointing medical university presidents is structured around three main themes: political preferences, university independence, and routine priorities. This framework includes 23 sub-themes that highlight the complexities and challenges inherent in the selection process.

- **The influence of political preferences**

The selection and appointment of university presidents have increasingly come under scrutiny, with many interviewees highlighting the pervasive role of political maneuvering in these processes. This influence is particularly pronounced among high-level executives, where political considerations often overshadow essential competencies required for leadership roles. In certain administrations, political allegiance has emerged as the primary criterion for selection, leading to the marginalization of meritocratic principles and the qualifications necessary for effective university governance.

Many interviewees criticized the current selection process as heavily influenced by political and personal interests, rather than being grounded in scientific merit or competence. Concerns were raised that political affiliations and regional considerations often overshadow qualifications, leading to appointments that do not prioritize academic or managerial capability. One participant articulated this issue clearly: “*In some cases, university presidents are appointed as political favors, with decision-makers accountable only to those who support their position, rather than to the academic community. This political influence, coupled with the absence of clear selection criteria, undermines the legitimacy of the process… In certain instances, certain pressure groups leverage the influence of the media to exert pressure on the Minister of Health to appoint a specific individual.*” (P24).

As one participant observed, “*In certain periods, we have witnessed the unhealthy and excessive use of political maneuvering to appoint managers characterized by political favoritism. This has rendered meritocracy and the principle of selecting the most qualified candidates insignificant in the face of political allegiance*” (P8). Another interviewee lamented, “*Unfortunately, in our country, the perception and interpretation mechanisms of some individuals are solely based on political inclinations and interests. This has led to a kind of affliction in the selection of individuals for the most academically rigorous environments, such as universities, resulting in long-lasting adverse consequences that we will grapple with for years to come*” (P29).

Despite this troubling trend, a consensus emerged among participants advocating for a return to merit-based selection practices. Many argued that university presidents should be distinguished figures in their respective fields, possessing the requisite skills and experience to lead effectively. The current reliance on political connections, they contended, undermines the integrity of academic institutions and stifles innovation.

Interviewees expressed concern that this environment is fostering a culture of isolation and silence within universities. The fear of dissenting opinions or criticism being perceived as hostility discourages open dialogue and the exchange of new ideas. As a result, institutions risk becoming stagnant, unable to adapt or evolve in an increasingly complex educational landscape. Ultimately, unless the selection process prioritizes merit over political affiliation, universities may continue to face significant challenges in fulfilling their vital roles in society.

- **The independence of universities**

The independence of universities has emerged as a pivotal theme among interviewees, many of whom assert that autonomous institutions are essential for enhancing efficiency and productivity. They contend that the current dependence of Iranian universities on external influences leads to conflicts of interest and operational inefficiencies. Consequently, there is a growing call for universities to embrace greater autonomy, particularly in the selection of university presidents. However, some experts caution that complete independence may inadvertently foster its own conflicts of interest, highlighting an ongoing debate regarding the delicate balance between institutional autonomy and governmental oversight.

Implementing necessary reforms to enhance university independence is perceived as a formidable challenge. Participants noted that overhauling administrative and financial processes will necessitate significant modifications to existing organizational structures and regulations, which are likely to encounter considerable resistance from both internal and external stakeholders.

As one interviewee articulated, “*Members of parliament, governors, and local clerics clearly interfere in the operations of universities, including the selection of university presidents. This undermines the integrity of the process*” (P27). Such interference is widely regarded as detrimental to the independence of academic institutions, where decision-making is often swayed by political figures.

The relationship between universities and governmental bodies also raises concerns. One participant remarked, “*In our country, universities are defined within the structure of the Ministry of Health. Given that science and scholarly work should inherently be free from any value-based interference, the independence of scientific institutions is at stake and must be prioritized. Universities must operate free from political pressures to preserve their academic integrity*” (P3).

Interviewees further suggested that the appointment of university presidents should be entrusted to a competent board of trustees, which would select candidates based on established criteria. The effectiveness of this approach hinges on the careful selection of board members; a weak or politically compromised board could undermine the entire process. There is a consensus that establishing clear criteria for both the board of trustees and the university president is crucial for ensuring effective appointments.

However, many participants criticized the current composition of boards of trustees in Iranian universities, arguing that political affiliations often overshadow the expertise necessary for effective governance. One interviewee lamented, “*Unfortunately, what we have observed from the board of trustees is that they cannot fulfill their roles in enhancing the performance of the university according to legal standards*” (P19). This perceived inadequacy raises significant concerns about the impact of favoritism and political influence on the academic standing and quality of universities.

While there is a strong desire among stakeholders for increased independence in Iranian universities, achieving this goal will require overcoming substantial challenges related to governance structures and political interference. The path forward necessitates a commitment to establishing robust criteria for leadership selection and ensuring that boards of trustees are composed of individuals with the requisite expertise to uphold academic integrity and institutional autonomy.

- **Gender and age dynamics**

The selection of university presidents within the Iranian medical sciences sector has been heavily influenced by longstanding traditions and existing power structures. As highlighted by interviewees, the predominance of male leaders and the age demographic of those in leadership positions reflect broader societal trends that may hinder diversity and innovation in academic governance.

The gender disparity in leadership roles is particularly striking. Many interviewees expressed concern over the lack of female presidents and the scarcity of leaders with non-clinical backgrounds, such as health policy or health management.

Also, the predominance of older leaders, often those over 50 raises questions about the inclusivity of decision-making processes and the potential for fresh perspectives in university governance. The lack of younger leaders may stifle innovation and adaptability, qualities that are increasingly essential in today’s rapidly evolving academic landscape.

As one participant articulated, “*It is rare to find a female university president or a university dean with non-clinical studies such as health policy or health management. Men have always been at the helm of medical sciences universities. Also rarely find a president with the age under 50*” (P15). This sentiment reflects a broader recognition of the barriers women face in ascending to leadership roles within academic institutions, particularly in fields traditionally dominated by male professionals.

This observation underscores the entrenched nature of professional networks that favor established figures within clinical specialties, often at the expense of broader representation.

One participant noted, “*The Medical Council and other professional associations within the medical sector, along with their lobbying efforts, are among the most influential players in regional health systems. Consequently, it is typical that, except in rare cases, university presidents are selected from specialists*” (P6).

These observations point to a critical need for reform in the leadership selection processes of Iranian medical universities. To foster a more inclusive environment, stakeholders must consider implementing policies that promote gender diversity and encourage the appointment of individuals with varied academic backgrounds. Such changes could enhance the effectiveness of university governance by incorporating a wider range of perspectives and experiences.

- **Assessment and evaluation**

The findings from document analysis and stakeholder interviews highlight a significant lack of a systematic framework and transparency in the selection of university presidents in Iran, particularly within medical universities. Although the process adheres to a general national approach, it is largely confined to a limited set of procedures and broad criteria.

As one interviewee noted, “*According to current regulations, a candidate for university president is recommended by the relevant minister and then presented to the Supreme Cultural Revolution Council, where their qualifications are reviewed by a supervisory committee. After deliberation, the council votes on the candidate, and the minister issues the appointment. However, this process lacks transparency and does not involve systematic consultation with faculty members or staff*” (P18).

Document analysis corroborates these concerns, revealing that while the minister is legally responsible for appointments, the Supreme Cultural Revolution Council must approve selected individuals. Historically, university presidents were elected by faculty members, indicating a troubling shift from participatory processes toward more centralized and politically driven appointments. As another interviewee pointed out, “The university president, similar to practices in many other countries, can be selected through the election of faculty members, students, or other staff within the university. The final decision may be subject to approval by the government or its representative, such as the Ministry of Health” (P13).

One manager emphasized a minister’s cautious approach to replacing university presidents, advocating for performance-based changes rather than blanket replacements, although this practice was not uniformly applied. Several interviewees suggested establishing independent committees at both the university and Ministry of Health and Medical Education (MoHME) levels to oversee the selection and evaluation of university president candidates based on evidence-based criteria. Furthermore, they recommended periodic evaluations of university presidents, their deputies, and managers at the end of each management term to ensure accountability and inform future appointments or re-selection.

“*The episodic nature of presidential selection is problematic; it is not regarded as a permanent task for the minister. Consequently, there is a perception that once it is done, it does not require ongoing attention. In reality, these selections are constant. Therefore, a structured plan with evidence-based criteria and performance assessments is essential. It is crucial that the selection process be integrated into the broader human resources framework of universities. Implementing succession planning would also be beneficial*” (P11).

Many participants strongly believed in the necessity of training and educational courses for newly appointed presidents. As one participant stated, “*It is important that appointed presidents undergo training related to the competencies required for university leadership to enhance their professional knowledge and skills*.”(P38)

Addressing these systemic issues in the selection process for university presidents is vital for fostering transparency, accountability, and academic integrity within Iranian medical universities. Implementing structured frameworks and continuous evaluation mechanisms can significantly enhance leadership effectiveness in these institutions (Table 5).

**Table 5:** Selection and appointment process of university presidents in Iran (results of the qualitative phase).

Themes	Sub-themes
Influence of political preferences	- Prevalence of political favoritism - Groups and personal interests - Prioritizing political connections - Impact on academic integrity - Role of pressure groups - Effects of political power - Stifling of innovation and dissent
The independence of universities	- Role of governance structures - The effects of Political Influence - Resistance from internal and external stakeholders - Interference by political figures - Setting Principles and oversight - Managing conflict of interests - Support for internal selection of university presidents
Assessment and evaluations	- Competency based criteria - Merit-based criteria - Prioritizing competencies over personal connections - Participatory to centralized appointments - Independent evaluation committee - Performance-based evaluation and accountability - Development of standards for reappointment - Training and education courses
Gender and age dynamics	- Age range in university leadership - The shortage of female university presidents - Priority of academic background - Full employment at the university

**Fig 4:**
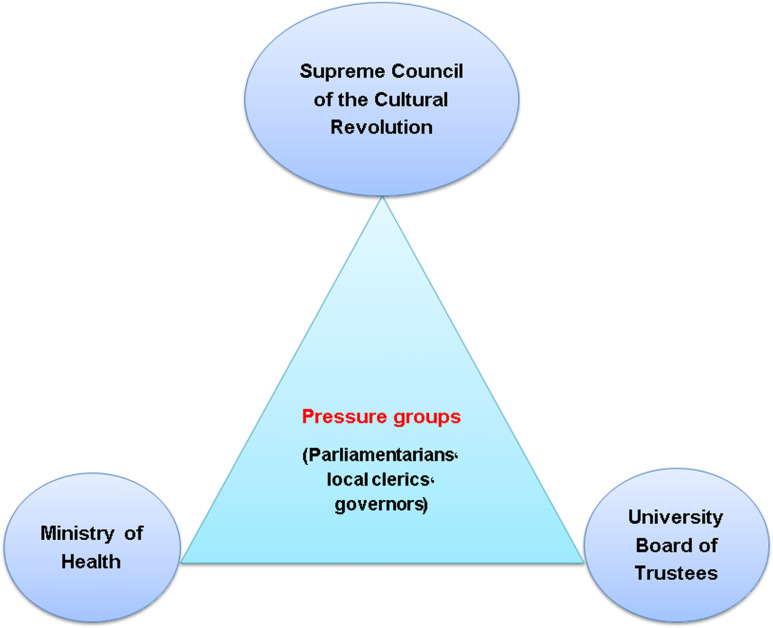
Influential stakeholders in the selection and appointment of university presidents in Iran (results of the qualitative phase).

Fig 4 shows influential stakeholders in the selection process and appointment of university presidents in Iran.

### Phase 3: Findings of the brainstorming

In this phase of the study, 12 key stakeholders included, the main characteristics of whom are shown in Table 6.

**Table 6:** Main characteristics of included participants in the brainstorming phase.

Organizational position	Gender		Working experience	Age	Number
	Female	Male	Year/ Average	Year/ Average	
Former minister of health and medical education		1	28 (4)	64	1
President of the university		3	24	57	3
Faculty member	2	4	22	52	6
Hospital manager		1	19	56	1
High-level employee of the Ministry		1	27	47	1
Total number of participants					12

**Policy recommendations for university governance:**

Through a comprehensive brainstorming session, four key policy recommendations emerged to enhance university governance and the selection of university presidents. By implementing these recommendations, universities can strengthen their governance structures, enhance the integrity of their leadership selection processes, and ultimately contribute to a more robust academic environment.

- **Increasing university autonomy**

To foster efficiency, productivity, and academic integrity, universities should be granted greater autonomy, particularly in the internal selection of their presidents. This increased independence can mitigate conflicts of interest that often arise from external political interference, thereby preserving the integrity of academic institutions.

- **Strengthening the role and position of university board of trustees**

One of the strategies for achieving university independence in Iran is to strengthen the role and position of university boards of trustees by revising the current regulations governing them. The use of clear and well-defined criteria in reviewing the membership, composition, and selection process of board members is also crucial in enhancing the board’s status. Board members should establish new connections within society to bridge existing gaps between universities and the community, fostering an institutionalized, purposeful, and impactful relationship. They should also play a role in securing and diversifying financial resources to promote university independence and growth. Additionally, the performance of university boards of trustees should be evaluated using appropriate assessment tools.

- **Developing an evidence-based framework for the selection of university presidents**

Strengthening the position of university boards of trustees and, consequently, moving toward university independence can have a significant impact on selecting qualified individuals for university leadership. In this process, it is essential to develop and implement a well-defined framework for selecting university presidents based on the latest domestic and international evidence. The framework should be designed in a way that ensures the maximum participation of university members, including faculty, non-faculty staff, and students, in the selection process. Additionally, reducing political interference, managing conflicts of interest, and enhancing transparency in the selection and appointment of university presidents should be key considerations within the designed framework.

- **Developing a framework for the continuation or dismissal of university presidents**

Periodic performance evaluation of university presidents during their tenure is one of the emphasized options. Accordingly, it is necessary to establish a framework based on evidence and transparent criteria for assessing their performance. The key dimensions of this framework should focus on accountability and responsibility as the basis for either continuation in office or dismissal. The evaluation framework must be comprehensive, encompassing multiple relevant dimensions of assessment.

**Fig 5:**
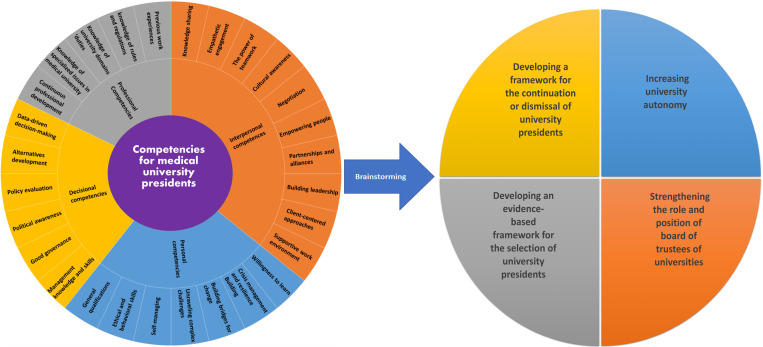
Key competencies for university presidents.

Fig 5 shows the key competencies found by the results of three phases of study.

## Discussion

This study employed a multifaceted approach to gather data on the essential competencies required for medical university presidents and the intricacies of their selection processes. The evidence suggests that implementing changes without a robust framework of policy, strategy, planning, and political acumen often results in superficial or inadequate outcomes. In the context of university leadership, high-level decision-making frequently occurs in isolation from long-term growth and development plans, yielding only transient effects.

The findings indicate that over successive presidential terms, limitations in macro-level vision and strategic approach have led to significant challenges within universities. It is imperative that proposed programs transcend isolated initiatives; they must adopt a holistic perspective that encompasses all dimensions of the university’s development as well as the broader developmental needs of the province and nation.

The findings of the scoping review phase highlight the variability and complexities inherent in presidential selection processes across diverse contexts, reflecting deeper issues of governance and accountability within higher education institutions. In Iran, policymaking does not adhere to a fully rational or bounded rational model; instead, it aligns more closely with the “garbage can” model of decision-making [55]. This model illustrates how various stakeholders primarily motivated by the preservation of their status, power, and interests interact within the decision-making landscape. A significant amount of time is consumed in efforts to persuade stakeholders who are often resistant to compromise their personal interests [56,57].

Furthermore, many challenges within the health system resemble an iceberg; only a small fraction of their underlying causes are visible. Beneath these surface issues lie vested interests that thrive in existing disorder, complicating efforts for meaningful change and perpetuating a façade of order amid chaos. This underscores the necessity for a more transparent and inclusive approach to governance in higher education, one that prioritizes long-term vision and collaborative engagement among all stakeholders.

Results of the current study indicated that the selection process for university presidents in Iran is widely regarded as opaque and heavily influenced by political considerations, highlighting an urgent need for reform. Stakeholders advocate for a more transparent, participatory, and criteria-driven approach to ensure that the most qualified candidates are appointed, thereby fostering motivation and trust within the academic community.

A critical issue in the selection of university presidents is the management of conflicts of interest [58]. Our study reveals that one of the most significant sources of conflict arises when university independence is undermined. This is particularly evident in cases where regional, ethnic, or political motivations drive interventions. Such influences can transform universities into instruments for personal agendas, diverting them from their core educational and research missions.

Drawing on experiences from developed countries, it is clear that preserving the independence and self-governance of universities is essential [59–61]. However, this does not imply that universities should operate without regulatory oversight or rely solely on the discretion of administrators and faculty members. A key consideration in this context is the standing and legitimacy of faculty members within the broader community, which may enable them to act independently of oversight or place them in decision-making roles [60,62].

To prevent university independence from becoming a source of conflict or ethical dilemmas, it is imperative to establish guidelines for managing conflicts of interest [58]. These guidelines should be developed through a careful analysis of the university’s structure, its internal and external relationships, and potential risk areas. Additionally, specific conflict-of-interest scenarios currently faced by the university must be identified to create detailed and practical guidelines that can be effectively implemented [58,63].

Evidence suggests that some policymakers, including Ministers of Health and senior administrators within executive agencies, appoint individuals to leadership positions based on alignment with specific policies rather than on merit. Such practices compromise the principles of competency and suggest a troubling prevalence of political favoritism. This underscores the necessity for a more equitable and principled approach to leadership selection in higher education [64].

In Land’s exploration of university leadership, the competencies required of a university president are categorized into three essential domains: managerial abilities (including educational, financial, and human resources management, as well as decision-making capabilities), leadership (the capacity to influence a diverse range of stakeholders from students to staff and policymakers), and personal qualities (such as integrity, trustworthiness, and patience) [65]. A pivotal consideration in evaluating the criteria for selecting university presidents is the recognition that possessing any given competency is preferable to lacking it entirely. For instance, an individual with robust financial management skills is inherently better suited to oversee a university than someone devoid of such expertise.

The primary challenge in the selection process arises when it becomes necessary to prioritize among various competencies. Moreover, prior managerial experience is of paramount importance. Evidence underscores the value of a career trajectory that allows university presidents to ascend through ranks, from department chair to university president [18,65]. Key managerial competencies such as planning, organization, problem-solving, and personnel motivation are critical for effective leadership. Additionally, strong communication and interpersonal skills are vital for accountability and success in leadership roles, particularly in interactions with subordinates, media, and board members. The ability to cultivate stable and transparent leadership practices emerges as a cornerstone of effective management [66,67].

Our findings indicate that many university presidents in Iran are underperforming in crucial areas, including strategic planning, community engagement, external stakeholder relations, and holistic systems-oriented thinking. The current criteria for selecting and appointing university presidents are inadequate and misaligned with the extensive responsibilities expected of them. This situation highlights the urgent need for well-defined, evidence-based measures to guide the selection process. Furthermore, the existing selection framework lacks a robust scientific foundation and fails to align with the diverse functions of these institutions, underscoring the necessity for a more transparent, systematic, and data-driven approach to appointing university leaders.

In summary, our findings advocate for a more nuanced understanding of the competencies required for university leadership, as well as the processes governing their selection. By prioritizing essential competencies and implementing a thorough evaluation process, universities can better position themselves to cultivate effective leadership capable of addressing contemporary challenges.

### Study strengths and limitations

This study benefits from a robust methodological framework that integrates global evidence derived from a scoping review of relevant literature with local insights obtained through document analysis and qualitative data collection. This dual approach has facilitated a nuanced understanding of the subject under investigation, highlighting the disparities between the current state of higher education leadership in Iran and practices observed in other countries.

We believe that the policy recommendations generated from this study are not only relevant to Iran but also applicable to other nations with comparable contexts.

It is important to note that terminology related to university leadership varies significantly across different countries, with terms such as president, chancellor, vice-chancellor, and dean each embodying distinct roles and responsibilities. In our scoping review, we made a concerted effort to encompass all relevant terminologies and examine the associated roles, ensuring a comprehensive analysis of leadership structures within higher education.

## Conclusion

The selection process for university presidents in Iran markedly contrasts with practices observed in other countries, primarily due to the pervasive influence of political dynamics and the interests of various stakeholders. This political entanglement often results in leadership changes at medical universities with each government transition, suggesting that political considerations frequently take precedence over qualifications and competencies in the selection of university presidents. This study showed that during various selection and appointment periods, political changes and pressure groups’ interests have had a significant impact on the management changes in universities, and with governments’ changes, managerial capabilities are overshadowed by political tendencies. To cultivate effective leadership, it is essential to establish a more comprehensive and transparent selection process that prioritizes both personal competencies and professional qualifications. Furthermore, the limited involvement of diverse societal groups in program development, coupled with a lack of visionary perspectives and coherent long-term strategies, often leads to the appointment of individuals driven by transient interests rather than enduring goals.

This study’s findings underscore the importance of transparency, evidence-based decision-making, and a systematic approach to the selection and appointment of presidents of medical universities. Implementing a systematic performance assessment for university presidents is crucial. Such evaluations should measure achievements against established goals and criteria, ensuring accountability while informing and enhancing future appointments. This approach will ultimately contribute to the advancement and integrity of higher education in Iran. Implementing these insights can enhance the integrity of the selection process and improve governance in medical education in Iran. The findings of this study can be applied by both medical and non-medical sciences universities in Iran as well as in other countries.

## Supporting information

S1 Table Primary search strategy in PubMed. (DOCX)

S2 Table List of included universities in the scoping review phase. (DOCX)

S3 File Interview Guide of the qualitative phase. (DOCX)

S4 Table The main characteristics of included studies in the scoping review phase. (DOCX)

S1 Checklist Preferred reporting items for systematic reviews and meta-analyses extension for scoping reviews (PRISMA-ScR) checklist. (DOCX)
