# Supplementary material for: Selection and appointment of presidents of medical universities in Iran: Bridging reality and ideal through global and local evidence
Source: PLoS One. 2025 Jun 24;20(6):e0326563. doi: 10.1371/journal.pone.0326563 (PMC12186901; doi:10.1371/journal.pone.0326563)
Supplement: S1 Table — (DOCX) [file pone.0326563.s001.docx]

# S1 Table:

# Primary Search Strategy in PubMed

Search conducted on September, 2024.

| **Search** | **Query** | **Records retrieved** |
| --- | --- | --- |
| #1 | (universit*[Title/Abstract]) OR (higher, education[Title/Abstract]) OR (school[Title/Abstract]) OR (college[Title/Abstract]) | 895,428 |
| #2 | (select*[Title/Abstract]) OR (recruitment[Title/Abstract]) OR (appointment[Title/Abstract]) | 2,660,657 |
| #3 | (president[Title/Abstract]) OR (lead*[Title/Abstract]) OR (board[Title/Abstract]) OR (committee[Title/Abstract]) OR (administrat*[Title/Abstract]) OR (trustee[Title/Abstract]) OR (dean[Title/Abstract]) OR (chancellor[Title/Abstract]) | 3,319,814 |
| #4 | (competenc*[Title/Abstract]) OR (skill[Title/Abstract]) OR (criteri*[Title/Abstract]) OR (qualification*[Title/Abstract]) | 1,070,268 |
| #5 | (medic*[Title/Abstract]) OR (health[Title/Abstract]) | 5,025,196 |
| #6 | #1 AND #2 AND #3 AND #4 AND #5 | **1526** |
| Without any limitation on date. | |  |

# Primary Search Strategy in Web of Knowledge

Search conducted on September, 2024.

((TI=universit* OR AB=universit*)) OR ((TI="higher, education" OR AB="higher, education")) OR ((TI=school OR AB=school)) OR ((TI=college OR AB=college))

AND
((TI=select* OR AB=select*)) OR ((TI=recruitment OR AB=recruitment)) OR ((TI=appointment OR AB=appointment))

AND

((TI=president OR AB=president)) OR ((TI=lead* OR AB=lead*)) OR ((TI=board OR AB=board)) OR ((TI=committee OR AB=committee)) OR ((TI=administrat* OR AB=administrat*)) OR ((TI=trustee OR AB=trustee)) OR ((TI=dean OR AB=dean)) OR ((TI=chancellor OR AB=chancellor))

AND
((TI=competenc* OR AB=competenc*)) OR ((TI=skill OR AB=skill)) OR ((TI=criteri* OR AB=criteri*)) OR ((TI=qualification* OR AB=qualification*))

AND
((TI=medic* OR AB=medic*)) OR ((TI=health OR AB=health))

# Primary Search Strategy in SCOPUS

Search conducted on September, 2024.

(TITLE-ABS(universit*)) OR (TITLE-ABS("higher, education")) OR (TITLE-ABS(school)) OR (TITLE-ABS(college))

AND
(TITLE-ABS(select*)) OR (TITLE-ABS(recruitment)) OR (TITLE-ABS(appointment))

AND

(TITLE-ABS(president)) OR (TITLE-ABS(lead*)) OR (TITLE-ABS(board)) OR (TITLE-ABS(committee)) OR (TITLE-ABS(administrat*)) OR (TITLE-ABS(trustee)) OR (TITLE-ABS(dean)) OR (TITLE-ABS(chancellor))

AND
(TITLE-ABS(competenc*)) OR (TITLE-ABS(skill)) OR (TITLE-ABS(criteri*)) OR (TITLE-ABS(qualification*))

AND
(TITLE-ABS(medic*)) OR (TITLE-ABS(health))

# Primary Search Strategy in PsycINFO

Search conducted on September, 2024.

(universit*.ti,ab.) OR ("higher, education".ti,ab.) OR (school.ti,ab.) OR (college.ti,ab.)

AND

(select*.ti,ab.) OR (recruitment.ti,ab.) OR (appointment.ti,ab.)

AND

(president.ti,ab.) OR (lead*.ti,ab.) OR (board.ti,ab.) OR (committee.ti,ab.) OR (administrat*.ti,ab.) OR (trustee.ti,ab.) OR (dean.ti,ab.) OR (chancellor.ti,ab.)

AND

(competenc*.ti,ab.) OR (skill.ti,ab.) OR (criteri*.ti,ab.) OR (qualification*.ti,ab.)

AND

(medic*.ti,ab.) OR (health.ti,ab.)
