# Supplementary material for: Selection and appointment of presidents of medical universities in Iran: Bridging reality and ideal through global and local evidence
Source: PLoS One. 2025 Jun 24;20(6):e0326563. doi: 10.1371/journal.pone.0326563 (PMC12186901; doi:10.1371/journal.pone.0326563)
Supplement: S2 Table — (DOCX) [file pone.0326563.s002.docx]

| Country | University/ Universities |
| --- | --- |
| USA | Stanford University  John Hopkins University  University of Cambridge  Yale University  Massachusetts Institute of Technology (MIT)  Colombia University |
| Canada | University of Toronto |
| England | Oxford University  King’s College London |
| Sweden | Karolinska Institutet |
| Japan | Kyoto University  Osaka University |
| Turkey | Istanbul University  Gazi University  Koc University |
| Germany | University of Hamburg  University of Bonn |
| Austria | University of Vienna |
| Netherlands | University of Amsterdam |

**S2 Table. List of included universities in the scoping review phase**
