# Supplementary material for: Selection and appointment of presidents of medical universities in Iran: Bridging reality and ideal through global and local evidence
Source: PLoS One. 2025 Jun 24;20(6):e0326563. doi: 10.1371/journal.pone.0326563 (PMC12186901; doi:10.1371/journal.pone.0326563)
Supplement: S3 File — (DOCX) [file pone.0326563.s003.docx]

**S3 File. Interview Guide of the qualitative phase**

This guide has been tailored to align with the specific job position of the interviewee, ensuring relevance and depth in the inquiries presented.

1. In your opinion, what characteristics and competencies are essential for an effective university president?
2. When making decisions, what criteria should a university president prioritize? To what extent is evidence-based decision-making feasible within this role?
3. Can you describe the process of selecting and appointing university presidents in our country?

- What are the strengths and weaknesses of this process?
- Which factors significantly influence this selection process?

1. Reflecting on your tenure as a university president or in other leadership roles, what experiences stand out as particularly significant?
2. What recommendations would you offer to policymakers in the higher education sector regarding the selection and appointment of university presidents?
3. Are there any additional topics or points you believe warrant discussion? Please elaborate.
