# Supplementary material for: Selection and appointment of presidents of medical universities in Iran: Bridging reality and ideal through global and local evidence
Source: PLoS One. 2025 Jun 24;20(6):e0326563. doi: 10.1371/journal.pone.0326563 (PMC12186901; doi:10.1371/journal.pone.0326563)
Supplement: S4 Table — (DOCX) [file pone.0326563.s004.docx]

**S4 Table. The main characteristics of included studies in the scoping review phase**

Data extractor: Neda Kabiri

Date of data extraction: November 12-20, 2024

| Author/date | Country | Aim | Study type/source- methodology | Setting/ University | Participants | Main results | |
| --- | --- | --- | --- | --- | --- | --- | --- |
|  |  |  |  |  |  | Selection process of university presidents | Competencies of university presidents |
| Gibney 2007 | USA | To describe the use of the analytical hierarchy process (AHP) in the dean selection process | Qualitative study | Business school |  | Analytical hierarchy process (AHP) | 1. Leadership:   - interpersonal/environmental skills: - environmental analysis skills (SWOT analysis) - candor and honesty - trustworthiness - vision - ability to develop a vision - ability to build consensus around the shared vision - an inclusive style of leadership   2. Access to outside resources and an ability to use them   - Internal focus - graduate students’ access to research databases, funding, employment opportunities, and facilities (e.g. computer hardware and software, and state-of-art classrooms) - External focus - the ability to raise funds from local businesses - the ability to increase alumni contributions to the university - the ability to obtain named endowments   3. Be appointed as a tenured full professor at the university |
| Khalili 2020 | Iran | To identify and validate the  leadership competencies required by university presidents in Iran | Descriptive survey (review: identify and survey: validate) | Iran, Tehran University, Allameh Tabatabai University and Kharazmi University | Faculty members of the 3 Universities | NS | Leadership competencies:   - Academic competence and academic credibility - Appointments away from political factions - Diversification of financial resources |
| Lavigne 2020 | Canada | To examine how the stated roles and qualifications of Canadian university presidents and provosts have evolved over the past thirty years | Document review | 22 Canadian universities | 53 job advertisements published by 22 universities  between 1987 and 2017 | Job advertisements | - An understanding of universities - Strong administrative skills - To a lesser extent, connection with either government or industry |
| Hassan 2008 | USA/ New York and Florida | To validate the competencies of presidents and trustee board chairpersons | PhD Thesis/ descriptive non-experimental | New York and Florida community  colleges | Presidents and trustee board chairperson of community  Colleges (n= 59) | NS | - Organizational Strategy - Community College Advocacy Communication - Professionalism - Collaboration - Resource Management - Inclusivity - Fundraising - Technology - Political sensitivity - Negotiation skills - Ethics   Character based competencies such as:   - Courage - Strength and wisdom - Legal skills   Interpersonal skills such as:   - Developing teams - Cultural competence - Authenticity - Managing stress - Using power wisely   Other skills:   - Institutional acumen - Strength to stand up to political pressure - Communication with sponsors - Respect employees |
| Adida 2009 | Belgium | To document the new version of Helios used in this election, the specifics of the UCL deployment, and the lessons learned in this deployment. | Descriptive | Universite catholique de Louvain (UCL) | 25,000 eligible voters | Helios Web-based open-audit voting system | NS |
| Kyamanywa 2021 | Uganda | To investigate the key leadership competencies required by deans of medical schools in Uganda | Qualitative cross-sectional study drawing on Grounded theory | Medical schools | 13 deans (9 of the 12 current deans and 4 former deans) | Election by the faculty members | 11 Leadership Competencies for Effective Deanship in 3 categories:   1. Personality  - Open to Learning and self- improvement  1. Education/training  - Medical expert - Medical educator - Research skills  1. Organizational skills  - People management - Work ethics - Financial and resources management - Communication and collaboration - Change management |
| Banaszak-Holl 1994 | USA | To assess turnover rate of medical school deans in the past five decades (1940-1990) | Review of published information from listings of American Medical Association and the Association of American Medical Colleges | Medical schools | 862 USA medical school deans | Average tenure for all deans equaled to 5.3 years with 55% of the individuals in office for four years or less.  Average tenure has decreased historically, from an average of 6.7 years in 1940s to an average of only 3.5 years in 1980s. |  |
| Wheat 2016 | USA | To understand the complex factors that influence women’s experiences in senior administrative roles | Qualitative study | Doctorate-granting universities | 14 women senior administrators (i.e., dean, vice president, provost, and presidents) | NS | 1. Participants’ pluralistic leadership styles and practices 2. The salience of gender in influencing women leaders’ perceptions and experiences 3. The role of women’s intersecting identities in shaping their leadership |
| Minton 2018 | USA | To examine the pathways, competencies, and preparation of community college presidents | PhD thesis/ Cross-sectional | Community colleges | 145 Community college leaders (70 presidents, 71 chief academic officers, and 4 other leadership positions) | An average of approximately 9.5 years in the president position | 1. Organizational Strategy 2. Resource Management 3. Communication 4. Collaboration 5. Community College Advocacy Professionalism 6. Adaptability 7. Entrepreneurship 8. Political Acumen 9. Technical Expertise 10. Fundraising 11. Personnel Management 12. Integrity 13. Teaching 14. Conflict Resolution 15. Change Management 16. Legal Expertise 17. Resource Development 18. Team Building |
| Alizadeh 2019 | Iran | To determine core competencies that can be improved through development program (trainable) for academic leaders in Iran University of Medical Sciences | Multi-method: a critical review and a modified Delphi | University of Medical Sciences | In the first and third rounds of Delphi: Experienced experts in management  and medical education  In the second  Round: vice-chancellors of education, deans of medical school, and head of department in Iran University of Medical Sciences | NS | Personal competencies:   1. System thinking 2. Punctuality 3. Role-model 4. Communication skill 5. Social intelligence 6. Emotional intelligence 7. Ethical 8. Continuous individual development 9. critical thinking 10. Full recognition of duties 11. Understanding the university environment and its structure and function 12. Understand the effective and up-to-date management practices of the university 13. Ability to respond to the change 14. The ability to effectively use information technology   Functional competencies:   1. Teamwork 2. Problem-solving skills 3. Responsiveness to organizational goals 4. Motivate, maintain morale, and support the faculty and staff 5. Conﬂict management 6. Encouraging and improving the innovation 7. Giving feedback and receiving constructive feedback 8. Ability to create coalition and change support |
| Al-Omari 2003 | Jordan | To assess the competencies required by presidents of universities | Cross-sectional | 3 Jordanian institution of higher education | 86 academic  administrators and 139 faculty members | NS | 1. International Culture, Vision, and Mind set 2. International Strategic Planning 3. International Human Resources 4. International Operations and Structure 5. International Learning |
| Enomoto 2007 | USA | To give an in-depth look at a dean’s selection and socialization process | Qualitative Case Study | A research-extensive, public institution, governed by a board of trustees and nationally accredited | A case study | Search and selection committee | NA |
| McLean 2019 | USA/ State of Louisiana | To identify and prioritize the essential leadership competencies for college presidents | PhD thesis/ qualitative research approach |  | 13 individuals | NS | 1. Fosters the development and creativity of learning organizations 2. Engages multiple perspectives in decision making 3. Learns from self-reflection 4. Sustains productive relationships with networks of colleagues 5. Applies analytical thinking to enhance communication in complex situations 6. Facilitates the change Process 7. Demonstrates resourcefulness 8. Demonstrates ability to diplomatically engage in controversial issues 9. Demonstrates negotiation skills 10. Seeks to understand human behavior in multiple contexts 11. Accurately assesses the costs and benefits of risk-taking 12. Facilitates effective communication among people with different perspectives 13. Demonstrates understanding of complex issues related to higher education 14. Responds appropriately to change 15. Presents self professionally as a leader 16. Communicates vision effectively 17. Communicates effectively 18. Expresses views articulately in multiple forms of communication 19. Demonstrates unselfish leadership 20. Learns from others 21. Does not take self too seriously 22. Works effectively with the media |
| Harvey 2011 | - | To examine the selection of deans from a theoretical perspective by employing reference point theory | Literature review | - | - | Search and selection committee |  |
| Noorshahi 2006 | Iran | To recognize competencies from the related literature in higher education and management | Document review and Aggregation Group Opinion using fuzzy technique | Iranian higher education institutes | 14 experts in Aggregation Group Opinion |  | Characteristics:  1. Creativity:   - The ability to look at issues from different angles than others. - Ability to find suitable and new solutions for problems   2. Behavioral coherence:   - Sticking to his words and that his actions match his words.   3. Adherence to moral principles   - Reputation for compliance with ethical principles   4. Being outstanding in terms of science   - Having the scientific rank of professor and good reputation and academic credit - Having a history of active membership in scientific-professional-specialized associations - History of chairing scientific associations   5. Being experienced in higher education and having motivation   - Having management experience at the department, faculty and other management levels of a university or university-like institutions for at least 5 years   Skills:  6. Change management   - Having the ability to give energy and motivate people and active listening - Developing and empowering and giving the possibility to use the ability to colleagues   7. Conflict resolution   - The ability to manage conflict - Getting to know the concepts of conflict management in human relations   8. Ability to take and receive financial resources   - The ability to find new financial sources - Encouraging institutions and organizations to financially support the university   9. Creating mutual trust   - Coordination in response   10. Strategic planning   - Familiarity with strategic planning techniques - Experience of participating in strategic planning and management training courses   11. Interpersonal and communication skills   - Having strong communication skills and establishing healthy relationships with others   12. Bargaining   - The ability to bargain and convince the other party   13. Negotiation   - Familiarity with negotiation techniques   Roles:  14. Representation and speaker   - The ability to play the role of representative within the organization and having the ability to create and build a coalition, understand and understand the opinions of others. - Having an open and receptive face and the ability to express attraction   15. Communicator   - Enjoying accurate human relations - Familiarity with properties and scientific foundations of organizational human behavior and relations   16. Crisis manager   - Familiarity with the basics and principles of crisis management and experience of participating in crisis management courses and the ability to solve short-term and urgent issues   17. Supervisor for the implementation of laws   - Knowing the laws and adhering to the implementation of the laws   18. Evaluation   - Getting to know evaluation techniques and methods and gaining experience in evaluating plans and programs |
| Rahimian 2019 | Afghanistan/ Kabul | To design and validate the competence model of the presidents of Kabul public universities | Mixed method- exploratory design | Public universities | Qualitative phase: 10 experts  Quantitative phase: 304 academic staff |  | Personal characteristics:   1. Interactive 2. Attractiveness 3. Committed 4. Motivator 5. Critic 6. Powerful 7. Well mannered   Theoretical knowledge:   1. Management knowledge 2. Scientific knowledge 3. Revolutionary 4. Monitoring and evaluation 5. Human resource management 6. Financial management   Practical skills:   1. Problem solving skills 2. Practical ability 3. Futuristic   Results of the qualitative phase:   1. Familiarity with information technology and communication 2. Familiarity with the rules and regulations and administrative structure 3. Familiarity with management and leadership theories in higher education 4. Familiarity with English language in management 5. Being experienced in higher education and being a member of the academic staff 6. Time management skills 7. Academic management experience 8. A history of success in previous jobs 9. Responsible and conscientious 10. Analytical and creative 11. Crisis and conflict management 12. Adherence to ethical principles 13. Risk taking power and adaptability 14. Management stability and negotiation and bargaining power 15. Having the necessary courage 16. Strategic planning and strategic vision 17. Change management |
| Yi 2016 | Korea | To mathematically consider the problem of simple random sample for composing the committee, and suggests an alternative method of composing committee which confirms more the variety of the university members | Review | A university that has selected the president candidate by the indirect election system | - | Search and selection committee | NS |
| Monks 2012 | USA | To examine job turnover among US university presidents from 2001–2006 | Secondary analysis of survey data sets | Data for 787 matched institutions that had valid presidential job durations, for the 2001 and  2006 survey years | 1,181 president’s responses in 2001, and 2,148 college and university president’s responses in 2006. | The average length of time in office (these are uncompleted spells in office) rose from 6.5 years in 2001 to 8.7 years in 2006. | NS |
| Al-Asfour 2021 | USA | To investigate the skills and competencies needed for candidates to be hired as a college or university president before the age of 40 | Qualitative study- a phenomenological approach | A four-year university, a four-year college, and a community college | 9 participants who obtained a presidency position before the age of 40 | NS | 1. Little to no mentoring at all, learn by either sinking or swimming; 2. Being in the right place, at the right time, with the right credentials; 3. Having the right education and experience matter for candidates seeking a college or a university president position; 4. Having the interpersonal skills and the ability to work with others |
| Rich 2008 | USA | To review the literature and resources for professional development of medical school executives in order to identify the characteristics proposed as relevant to medical school deanship | Literature review | North  American medical schools | 33  articles |  | Management Skills:   1. Institutional Assessment 2. Negotiation and conflict management 3. Change management 4. Communication with diverse audiences 5. Strategic planning 6. Financial stewardship 7. Fundraising 8. Team-building 9. Recruitment and retention of talent   Leadership Skills:   1. Visioning 2. Maximizing values 3. Knowing self 4. Mentoring 5. Building constituency 6. Making sense of experience 7. Challenging experience   Knowledge:   1. Academic medical center governance 2. Legal and regulatory issues 3. Challenges and expectations of clinicians 4. and scientists 5. Process of medical education   Attitudes:   1. Commitment to the success of others 2. Appreciation of institutional culture 3. Patience with process |
| Liu 2019 | China | To understand University President Leadership in China | Review |  | 175 articles | Search and selection committee | 1. Ability in management and decision-making 2. The best scholars in terms of teaching and research 3. Having doctoral degree 4. focus on implementing reforms in practice 5. Being energetic and accept new thinking, creative, and have innovation 6. Have overseas experience 7. Having a comprehensive understanding of education 8. Personal qualities such as moral characteristics 9. Good insight 10. Organizational capability 11. Ability to concentration |
| Buckley 2014 | USA | To review the role of deans in US academic medical centers | Commentary- review | Medical schools | NS | NS | 1. Scholarship 2. Administrative acumen 3. Skill in recruitment 4. Effectiveness as a departmental and institutional administrator 5. Visionary perspective collectively 6. Integrity 7. Trust 8. Financial understanding 9. Decisiveness 10. Professional maturation 11. Communication |
| Spendlove 2007 | UK | To investigate the role of the Pro-Vice-Chancellor, Rector, or Principal of a university, and the competencies (attitudes, knowledge and behavior) that are needed for effective leadership in higher education. | Qualitative study | 10 UK universities representative of the sector | 10 Pro-Vice-Chancellors: five from post-1992 universities (former polytechnics), and five from pre-1992 universities. |  | 1. Openness 2. Honesty 3. The need to consult others 4. The ability to listen, negotiate and persuade 5. The ability to think broadly/strategically 6. To engage with people 7. Academic credibility 8. Knowledge of academic life 9. Knowledge of how the university system works 10. Knowledge of the “academic coal face” 11. Knowledge of the differences between a good and a bad university 12. An understanding of academic processes 13. Team building and effective communication |
| Tobias 2013 | USA | To explore the relationships between the leadership attributes, skills, and career paths of college and university presidents in the United States | A PhD dissertation in Georgia University.  Non-experimental research- survey research | U.S. college and university presidents | 410 college chief executive officers | NS | 1. Provide vision 2. Demonstrate commitment 3. Create an environment of trust 4. Lead by example 5. Possess perseverance 6. Honest and fair 7. Demonstrate personal integrity 8. Respect others 9. Exhibit character 10. Lead the team of executive officers 11. Serve as the “face” of the institution 12. Recruit key leadership 13. Problem solve 14. Serve as the “face” of the institution 15. Being visible 16. Listen to others 17. Possess strong written communication skills 18. Act strategically 19. Being accountable to the governing body of the institution 20. Being a strategic planner for the institution 21. Work with a variety of constituents 22. Serve as a change agent 23. Fund raising 24. Conflict management skills 25. Serve as a political lobbyist |
| Turner 2005 | USA- Texas | To determine if chancellors, boards of trustee/regent members, board of trustee/regent chairs and presidents deem there is a communal set of traits, skills, training and temperaments, which an individual seeking to become a community college president must possess to lead a community college | A PhD dissertation in The University of Texas at Austin  Cross sectional study | 51 Texas community college districts | 94 participants (37 Board of  Trustee/Regent Member, 13 Board of  Trustee/Regent Chairperson, 4 chancellors, and 40 Presidents) | NS | 1. Ability to accept personal responsibility 2. Ability to effectively lead the leadership team 3. Ability to build trust 4. Personal code of ethics 5. Ability to influence and motivate people 6. Ability to make and stand by tough decisions 7. Commitment to learning 8. Ability to control personal conduct 9. Ability to build consensus 10. Commitment to students 11. Ability to maintain core values 12. Ability to incorporate a culture of teambuilding 13. Commitment to teaching 14. Verbal communication 15. Ability to delegate authority 16. Ability to demonstrate consistent judgment |
| Hromas 2018 | USA | To examine the leadership characteristics that a chair thought were important for a medical school dean | Survey | 41 medical schools | 90 medical school  chairs | NS | 1. Accountability 2. Crisis management 3. Integrity between words and deeds 4. To define a strategic vision 5. Providing good feedback 6. Admitting errors 7. Open discussion of complex or awkward topics 8. Skill in improving relations with the teaching hospitals 9. Financial acumen (the least important skill a chair thought a dean should hold) |
